# Subcutaneous nodules revealing systemic sarcoidosis: a case report

**DOI:** 10.11604/pamj.2018.31.94.16650

**Published:** 2018-10-08

**Authors:** Nabil Tiresse, Hajar Benataya

**Affiliations:** 1 Military Hospital of Instruction Mohamed V, Department of Pulmonology, Rabat, Morocco

**Keywords:** Lupus pernio, subcutaneous nodules, épithélioide granuloma, corticoids, antimalarial drugs

## Image in medicine

57-year-old patient, treated for high blood pressure, never treated for tuberculosis and without toxic habits, having presented for one month a cutaneous lesions prevailing on the two upper limbs and on the face, painless, without pruritus, associated to redness eye and dyspnea stage II of the mMRC; the clinical examination revealed papules, subcutaneous nodules of 0.5cm on average and not exceeding 1cm for the most voluminous on the arms and forearms and on the face and the left eyelid, a lupus pernio is also present on the nose. Some lesions have presenting inflammatory signs. The pathological anatomy study of a skin biopsy revealed a granuloma with epithelioid and giant cells without caseous necrosis, suggesting the diagnosis of sarcoidosis first. A chest x-ray showed mediastinal lymphadenopathy and bilateral interstitial syndrome confirmed on thoracic computed tomography. The exploration of respiratory function reveals a restrictive ventilatory disorder supporting pulmonary parenchymal involvement. Ophthalmological examination shows anterior uveitis. The angiotensin converting enzyme assay is high and the tuberculin skin test reveals an anergy. The patient was treated by corticosteroids; synthetic antimalarials will be prescribed when no response or insufficient response happens. Subcutaneous sarcoidosis, also known as Darier-Roussy, and lupus pernio, are uncommon but specific of sarcoidosis. Their presence is often associated with systemic involvement, particularly thoracic.

**Figure 1:**
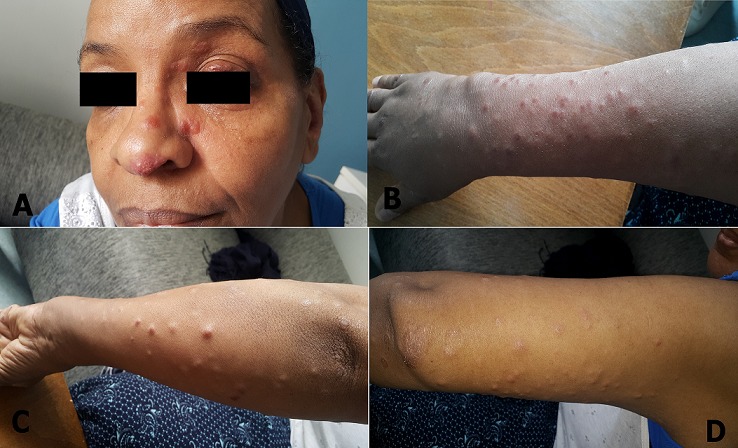
image showing a specific aspect of cutaneous involvement in sarcoidosis (A) lupus pernio at the nose; (B) subcutaneous nodules on the posterior aspect of the forearm; (C) subcutaneous nodules on the lateral and anterior aspect of the forearm; (D) subcutaneous nodules on the posterior aspect of the arm

